# Measuring disease activity and severity in clinical trials and the clinic: same or different?

**DOI:** 10.1186/ar4222

**Published:** 2013-07-11

**Authors:** Michelle Petri

**Affiliations:** 1 Professor of Medicine, Johns Hopkins University School of Medicine, Baltimore, Maryland, USA

## 

Standardized criteria to classify systemic lupus erythematosus (SLE) disease activity provide a well-defined methodology to accurately assess SLE. These criteria were recently revised to reflect new knowledge of SLE, both clinical and immunologic. Although developed to classify SLE, these criteria can help with the diagnosis in clinical practice. Research has also provided additional evidence of elevated morbidity and mortality associated with high-dose prednisone therapy, defined as greater than 6 mg/day. At these doses, significant increases in organ damage and cardiovascular events occur. Use of NSAIDs is also known to raise cardiovascular risk. Better options include intermittent intramuscular triamcinolone and hydroxychloroquine. The question of how often to monitor patients with stable SLE is confounded by the many variables showing clinical activity and their average frequency of occurrence. Nevertheless, the evidence suggests that 3 months is the ideal time for follow-up testing. Although there is no confirmed technical standard for measuring SLE disease activity, two primary instruments are available in the clinic: the Physicians Global Assessment and the SELENA-SLEDAI instrument. Each has varying degrees of ability to detect changes in disease activity as well as clinical usefulness. In clinical trials, outcomes are measured by the SLE Responder Index, an instrument that defines response based on criteria from SELENA-SLEDAI, Physicians Global Assessment, and British Isles Lupus Assessment Group index. In clinical practice, responses are primarily based on improvements in disease activity, especially reduced flares. Improvements in serology also matter, as patients with high anti-DNA or low complement are twice as likely to flare during the next year. Quality-of-life criteria are important to patients. Overall, the goal is to accurately measure clinical improvement in the patient's SLE disease activity.

Additional file 1 contains an edited transcript of the full presentation.

## Supplementary Material

Additional file 1 Click here for file

