# Supplementary material for: Measuring disease activity and severity in clinical trials and the clinic: same or different?
Source: Arthritis Res Ther. 2013 Jul 11;15(Suppl 2):A4. doi: 10.1186/ar4222 (PMC3891553; doi:10.1186/ar4222)
Supplement: Additional file 1 [file ar4222-S1.pdf]

Presentation

Measuring disease activity and severity in clinical trials and the clinic: same or different?

Michelle Petri

Professor of Medicine, Johns Hopkins University School of Medicine, Baltimore, Maryland, USA

Abstract

Standardized criteria to classify systemic lupus erythematosus (SLE) disease activity provide a well-defined methodology to accurately assess SLE. These criteria were recently revised to reflect new knowledge of SLE, both clinical and immunologic. Although developed to classify SLE, these criteria can help with the diagnosis in clinical practice. Research has also provided additional evidence of elevated morbidity and mortality associated with high-dose prednisone therapy, defined as greater than 6 mg/day. At these doses, significant increases in organ damage and cardiovascular events occur. Use of NSAIDs is also known to raise cardiovascular risk. Better options include intermittent intramuscular triamcinolone and hydroxychloroquine. The question of how often to monitor patients with stable SLE is confounded by the many variables showing clinical activity and their average frequency of occurrence. Nevertheless, the evidence suggests that 3 months is the ideal time for follow-up testing. Although there is no confirmed technical standard for measuring SLE disease activity, two primary instruments are available in the clinic: the Physicians Global Assessment and the SELENA-SLEDAI instrument. Each has varying degrees of ability to detect changes in disease activity as well as clinical usefulness. In clinical trials, outcomes are measured by the SLE Responder Index, an instrument that defines response based on criteria from SELENA-SLEDAI, Physicians Global Assessment, and British Isles Lupus Assessment Group index. In clinical practice, responses are primarily based on improvements in disease activity, especially reduced flares. Improvements in serology also matter, as patients with high anti-DNA or low complement are twice as likely to flare during the next year. Quality-of-life criteria are important to patients. Overall, the goal is to accurately measure clinical improvement in the patient's SLE disease activity.

CLASSIFICATION OF SYSTEMIC LUPUS ERYTHEMATOSUS

Surveillance of patients with systemic lupus erythematosus (SLE) requires accurate classification of the disease. To ensure a consistent definition of SLE, the Systemic Lupus International Collaborating Clinics (SLICC) group recently revised the American College of Rheumatology (ACR) SLE classification criteria based

Disclosures

About this presentation
This presentation was developed from an audio transcript of Dr. Petri's presentation at the "Treatment of SLE: Bridging the Gap from Clinical Trials to Practice" symposium held during the Annual Congress of the American College of Rheumatology on November 11, 2012.

The transcript was formatted and edited by Cleveland Clinic and BioMed Central staff for clarity and conciseness, and was then reviewed, revised, and approved by Dr. Petri.

Sponsorship
The Cleveland Clinic Foundation Center for Continuing Education acknowledges an educational grant for support of this activity from Human Genome Sciences.

Accreditation
The Cleveland Clinic Foundation Center for Continuing Education is accredited by the Accreditation Council for Continuing Medical Education to provide continuing medical education for physicians. The Cleveland Clinic Foundation Center for Continuing Education designates these (4) enduring activities for a maximum of **1.0** *AMA PRA Category 1 Credit*™. Physicians should claim only the credit commensurate with the extent of their participation in the activity. Participants claiming CME credit from this activity may submit the credit hours to the American Osteopathic Association for Category 2 credit.

To claim CME credit, visit www.ccfcme.org/SLECMEsupplement. CME credit may be obtained upon reading all four CME-certified presentations online.

Author disclosures
In accordance with the Standards for Commercial Support issued by the Accreditation Council for Continuing Medical Education (ACCME), The Cleveland Clinic Foundation Center for Continuing Education requires resolution of all faculty conflicts of interest to ensure CME activities are free of commercial bias. Dr. Petri has indicated that she may have relationships which, in the context of her presentation, could be perceived as a potential conflict of interest. Dr. Petri has received fees from HGS, Glaxo, MedImmune, UCB, TEVA, Pfizer, and Anthera for consulting and clinical trials.

All other planners, CME staff, and content reviewers, have no relevant financial relationships to disclose.

This presentation has not been subject to peer review. The statements and opinions expressed herein are those of the author, who bears full responsibility for the content of this presentation.

on regression analyses of SLE and control patients [1]. The SLICC used a large set of clinical scenarios and stringent methodology in their revisions and then subjected the criteria to validation.

The revised criteria provide two options for classifying SLE:

- Patient has at least four criteria: one has to be a clinical criterion and one an immunologic criterion.

  or:

- Patient has lupus nephritis diagnosed by kidney biopsy demonstrating immune complex-mediated glomerulonephritis compatible with lupus nephritis in the setting of antinuclear antibodies or anti-DNA.

The revisions kept the rule of needing to meet at least four criteria, but the SLICC now requires that at least one be a clinical criterion and one be an immunologic criterion. The SLICC also revised the list of 17 clinical and immunologic criteria. Rationales for changes are described below.

Clinical criteria

The revised clinical criteria classification lists 11 criteria (Table 1). The first criterion – acute and subacute cutaneous lupus – is new. Subacute lupus was omitted in the previous ACR criteria. The second criterion – chronic cutaneous lupus – replaces discoid rash. This term includes discoid lupus as well as all the other kinds of chronic cutaneous lupus disease such as tumid lupus and verrucous lupus.

The third criterion – oral and/or nasal ulcers – is the same. The fourth criterion – nonscarring alopecia – has been resurrected from the American Rheumatism Association criteria for SLE. It is typical of lupus because of the two hair findings of lupus: fragile (breaks off with minor trauma) and thinner than normal (scalp shows through). If the patient has scarring alopecia, that counts under chronic cutaneous lupus.

The fifth and sixth criteria – arthritis and serositis – are the same. The renal criterion (seventh criterion) is for patients who have not had a kidney biopsy; a spot urine protein:creatinine ratio of 500 mg/24 hours or more can be used.

The neurologic criterion (eighth criterion) is improved. Previously, it was limited to psychosis and seizures. The new definition (for example, seizures, psychosis, mononeuritis multiplex, myelitis, peripheral or cranial neuropathy, cerebritis) is more representative of neurologic lupus. In fact, seizures and psychosis are rarer now in SLE than when the ACR criteria were devised.

Hematologic anemia manifestations have been separated into three criteria: hemolytic anemia (criterion nine), leukopenia or lymphoma (criterion 10), and thrombocytopenia (criterion 11). In the previous classification, they were all lumped together.

Table 1. Revised Systemic Lupus International Collaborating Clinics/American College of Rheumatology classification system: clinical criteria

Clinical criteria	
1.	Acute/subacute cutaneous lupus
2.	Chronic cutaneous lupus
3.	Oral/nasal ulcers
4.	Nonscarring alopecia (diffuse thinning or hair fragility with visible broken hairs) in the absence of other causes such as alopecia areata, drugs, iron deficiency, and androgenic alopecia
5.	A. Inflammatory synovitis with physician-observed swelling of two or more joints
	OR
	B. Tender joints with morning stiffness
6.	Serositis
7.	A. Renal: urine protein/creatinine (or 24-hour urine protein) representing at least 500 mg of protein/24 hours
	OR
	B. Red blood cell casts
8.	Neurologic: seizures, psychosis, mononeuritis multiplex, myelitis, peripheral or cranial neuropathy, cerebritis (acute confusional state)
9.	Hemolytic anemia
10.	A. Leukopenia (<4,000/mm^3 at least once)
	OR
	B. Lymphopenia (<1,000/mm^3 at least once)
11.	Thrombocytopenia (<100,000/mm^3 at least once)

Immunologic criteria

Table 2 presents the revised immunologic criteria. The first criterion – antinuclear antibody test results – stayed the same. The second criterion – anti-DNA antibody level – has been revised to address frustrations with the ELISA testing results. Low titers from ELISA testing had mistakenly identified SLE in many patients who did not have SLE. The criterion now requires a stricter cutoff for ELISA anti-DNA antibody levels — it has to be twice the upper limit of normal to meet this criterion.

Presence of anti-Sm antibodies is now a separate criterion (third criterion). The antiphospholipid antibodies are in the fourth criterion. This has been expanded to allow IgG, IgM, or IgA isotypes for anti-β_2 GPI and anticardiolipin antibodies, which reflects new insights into the role of IgA isotypes in SLE. Low complement is the fifth criterion, reflecting its importance in SLE pathogenesis.

Criterion number six is the direct Coombs test. The regression analysis showed that the direct Coombs test detects a specific antibody for SLE, and the great majority of patients with positive results will never develop autoimmune hemolytic anemia. Nevertheless, this test is not counted if the patient has hemolytic anemia.

Additional notes on the revisions

One should note that these criteria were developed for classification of SLE; however, in clinical practice, they also can help with the diagnosis.

This presentation has not been subject to peer review. The statements and opinions expressed herein are those of the author, who bears full responsibility for the content of this presentation.

Table 2. Revised Systemic Lupus International Collaborating Clinics/American College of Rheumatology classification system: immunologic criteria

Immunologic criteria
1. Antinuclear antibodies above laboratory reference range
2. Anti-dsDNA above laboratory reference range (except twice above laboratory reference range for ELISA)
3. Anti-Sm antibodies
4. Antiphospholipid antibody positivity as determined by any of the following:
A. Positive test for lupus anticoagulant
B. False-positive test for syphilis
C. Anticardiolipin antibody level (IgA, IgG, or IgM) at least twice normal or medium-high titer
D. Positive test for anti-β_2 glycoprotein I (IgA, IgG, or IgM)
5. Low complement
A. Low C3
B. Low C4
C. Low CH50
6. Direct Coombs test in absence of hemolytic anemia

Some items were left out of the criteria that are going to become more important. One is the role of anti-C1q in lupus. This is important in terms of prognosis because it predicts patients who will develop lupus nephritis, as does anti-DNA and low complement. The greater role of anti-C1q is going to be following patients with lupus nephritis, because it is the only serologic test that correlates with disease activity in individual patients over time. There may be US Food and Drug Administration-approved quality anti-C1q assays that can be used in clinical practice.

PREDNISONE RISKS

As a therapy for patients with SLE, prednisone presents a clinical challenge because of its severe toxicities. It is especially toxic in high doses, which have been shown to significantly increase morbidity. In clinical practice, high doses should be defined as 6 mg/day rather than 0.5 mg/kg.

Toxicity claims are based on a considerable body of evidence from prospective studies. In the Hopkins Lupus Cohort [2], a cohort analysis of 525 patients with SLE, results showed that doses of 6 mg/day and above increased the risk of permanent organ damage by 50% (Table 3). However, some lupus nephritis regimens use doses >18 mg/day. At that dose, the odds for permanent organ damage are 2.5-fold increased.

This cohort also showed that prednisone increases cardiovascular (CV) events. At doses ≥10 mg/day, the CV event risk increased by 2.5-fold (P = 0.0002) [3]. At 20 mg/day, the risk increased to fivefold. This analysis was particularly rigorous because it adjusted for both the disease activity for which the prednisone was prescribed and for traditional CV risk factors. Because CV disease remains the major cause of late death in lupus patients, it is appropriate to conclude that these prednisone doses can contribute to mortality.

Table 3. Effect of prednisone on organ damage

Prednisone average dose	Hazard ratio
>0 to 6 mg/day	1.16
>6 to 12 mg/day	1.50
>12 to 18 mg/day	1.64
>18 mg/day	2.51

Adapted from [2].

These increased risks are not restricted to patients with SLE; it has been proven in other diseases, such as chronic obstructive pulmonary disease. These all point to the conclusion that prednisone can increase mortality.

Alternatives to prednisone

Not all patients with SLE have to be given chronic prednisone therapy, as there are well-documented therapeutic options. The Flares in Lupus: Outcome Assessment Trial showed that intramuscular triamcinolone, another corticosteroid, is an effective option [4]. This randomized clinical trial recruited patients with SLE who presented with mild to moderate flares. These were patients whose disease was not life-threatening.

Instead of increasing the prednisone dose and having patients return in 1 to 3 months, subjects were randomized to a short burst of two treatment options: oral prednisone with a rapid tapering (medrol dose pack for a week); or a single intramuscular dose of triamcinolone (100 mg). This trial used patient-reported outcomes, a rational choice because patients know when their symptoms improve (for example, rash, arthritis, fever).

Table 4 shows that at day 1 most patients receiving triamcinolone already felt better (approximately 70% reported partial improvement). At 4 weeks, their health status was very good.

From my viewpoint, not a single patient in this trial required additional therapy. If any patients had flared again at 1 month, this would have been a sign that they needed additional immunosuppressive therapy.

NSAID use and cardiovascular damage

In addition to eliminating prednisone use, practitioners also need to reduce use of NSAIDs, based on evidence linking chronic exposure to several toxicities. Data presented at the 2012 ACR meeting show that NSAID use significantly increases the risk of CV events in patients with mild to moderate SLE (hazard ratio, 1.66; P <0.03) [5]. This should not be a big surprise because the US Food and Drug Administration requires NSAID labeling to warn of the increased risk of CV disease. Additionally, there are known issues of nephrotoxicity associated with NSAID use in patients with lupus nephritis.

Treatment: hydroxychloroquine

Practitioners do have an option for safe, long-term background therapy in patients with SLE – hydroxy-

This presentation has not been subject to peer review. The statements and opinions expressed herein are those of the author, who bears full responsibility for the content of this presentation.

Table 4. Flares in Lupus: Outcome Assessment Trial: comparison of oral methlyprednisolone versus intramuscular triamcinolone

		Methylprednisolone (%)	Triamcinolone (%)
Complete improvement	1 week	8.3	8.6
	2 weeks	20.8	12.5
	3 weeks	20.8	30.4
	4 weeks	25.0	34.7
Partial improvement	Day 1	41.6	69.5
Health status	4 weeks	66.6	73.9

Adapted from [4] with major changes.

chloroquine (HCQ). In clinical trials, this antimalarial agent has shown efficacy for improving several SLE outcomes:

- *Reduced flares.* In a landmark Canadian study, HCQ reduced flares by 50% [6]. Although this study only enrolled 47 patients, results had enough statistical validity to change practice. When something works, it does not always have to be shown in a huge trial.
- *Reduced organ damage.* The LUMINA Study Group showed that HCQ prevented renal damage, but there were also data on prevention of central nervous system damage [7].
- *Reduced CV risks.* In addition to data showing reduced low-density lipoprotein cholesterol with HCQ therapy [8,9], it has also been shown to reduce thrombosis risks [10,11]. In addition, data were presented at the 2012 ACR meeting showing that HCQ reduces both arterial and venous thrombosis, particularly in antiphospholipid-positive patients with SLE.
- *Improved survival.* Several studies have documented improvements in mortality rates associated with HCQ therapy [12].
- *Improved response rates to mycophenolate mofetil therapy.* Data from the Hopkins Lupus Cohort found adding HCQ to mycophenolate mofetil tripled complete renal remissions rates in patients with lupus nephritis [13].

MONITORING SYSTEMIC LUPUS ERYTHEMATOSUS

Timing of follow-up

How often to schedule follow-up visits in a patient with stable SLE is an important clinical practice question. Data in Table 5 show that during a 2-year period one in four patients will have a silent symptom that can only be detected by laboratory tests [14]. This indicates that 3 months is the ideal time interval for routine follow-up clinical and laboratory tests.

Managing chronic fatigue

Chronic fatigue is the most common symptom in patients with SLE, affecting between 50 and 80% of patients [15]. In most patients, however, chronic fatigue is not caused by active lupus. Instead, their chronic fatigue is highly correlated with fibromyalgia, pain, depression, sleep abnormalities, and poor quality of life [16,17]. Fibromyalgia, for example, is seen in approximately 30% of patients with lupus [18]. Thus, prescribing medications for lupus will not address this complaint, and practitioners should focus efforts on treating the associated diseases that may be causing the fatigue.

One option for treating fatigue in these patients is use of tai-chi exercises. Their efficacy was shown in a small but well-controlled trial that found clinically significant, durable improvement in fatigue scores after 12 weeks. These scores increased out to 24 weeks[19]. This supports recommending tai chi to all lupus patients with fibromyalgia.

Furthermore, exercise in general has been shown to be beneficial in lupus patients [20]. At 12 weeks, patients who exercised had significantly better fatigue scores than the controls (48% vs. 16%; $P = 0.02$). These improvements were maintained to 3 months in patients who continued to exercise. These findings support recommending aerobic exercises to all eligible patients with SLE to manage fatigue.

MEASURING DISEASE ACTIVITY

Although there is no confirmed technical standard for measuring SLE disease activity, several instruments are available. Their clinical usefulness depends on several factors related to user friendliness and complexity of the test as well as their clinical accuracy.

Physicians Global Assessment

The Physicians Global Assessment tool uses a visual analog scale to measure disease activity both globally and in individual organ systems. The tool is relatively simple and quick to complete. Research has found it to be clinically relevant [21,22] and to correlate well with other disease indices [23]. Having the tool preprinted on an encounter form makes it easy for practitioners to use. Having visual scales makes it easy for practitioners to quickly assess which organs have been active. In general, having rheumatologists complete the Physicians Global Assessment tool at every office visit provides a degree of reliability for evaluating disease activity. However, the tool does have limitations, leading to some disagreement among lupus practitioners regarding its clinical importance.

This presentation has not been subject to peer review. The statements and opinions expressed herein are those of the author, who bears full responsibility for the content of this presentation.

Table 5. Detection of new clinical activity in systemic lupus erythematosus

Variable detected	Number of visits with new variable (*n* =173)	Number of patients with ≥1 visit with new variable (*n* = 127)
Cast	16	16
Hematuria	10	9
Proteinuria	15	15
Low complement	55	45
DNA antibodies	36	32
Thrombocytopenia	8	7
Leukopenia	7	7
Serum creatinine	9	8
Hemoglobin	6	6

Frequency of new isolated variables of interest in 515 patients, ≥18 months of follow-up. Adapted from [15].

SELENA-SLEDAI

The SELENA-SLEDAI (Safety of Estrogens in Lupus Erythematosus National Assessment – SLE Disease Activity Index) instrument is a validated disease activity index that lists 24 lupus manifestations. It meets the requirement of being easy and quick to complete – one simply checks the box for parameters that are present at the time of visit or have occurred in the previous 10 days. The checks also make it easy for practitioners to assess the results.

For practitioners, the SELENA-SLEDAI tool offers several positive features:

- The index only scores parameters that are present and attributable to SLE; partial changes in disease activity are not scored.
- Items are weighted with scores ranging from 1 to 8; more serious effects involving organs such as renal or the central nervous system have higher weights.
- The index sets a high threshold for responses; it is not easily triggered by minimal variations in disease activity.
- Score reduction requires complete elimination of disease signs and symptoms, or resolution of laboratory abnormalities.

This SELENA-SLEDAI instrument has limitations in clinical practice:

- The index does not track partial changes in disease activity (on the SLEDAI, it is either present or absent); thus, practitioners cannot determine whether symptoms have improved or worsened.
- The composite score cannot distinguish patients with multiple mild manifestations from those with fewer but more severe features.
- Improvement in one organ may be offset by new involvement in another organ.

Clinically, SELENA-SLEDAI is useful for identifying a patient's global score, but not for tracking partial improvement.

Pronunciation note

In clinical practice situations, SLEDAI should be pronounced 'slee-day' rather than 'slee-die.' Patients who hear 'slee-die' may misconstrue it as an instrument that predicts mortality. Thus, it is better to pronounce it 'slee-day.'

SLE Responder Index response criteria

Clinical trials

Do outcome measures used in clinical trials of patients with SLE have relevance for clinical practice? Realistically, the SLE Responder Index (SRI) – used to measure outcomes in SLE clinical trials – does not provide a practical option for clinical practice because it is not simple to use. For example, the SRI used in phase III trials of belimumab required responders to meet criteria from three tools: SELENA-SLEDAI, British Isles Lupus Assessment Group index, and Physicians Global Assessment (see Table 6).

The most important requirement was the four-point reduction on the SELENA-SLEDAI. This can have clinical importance for measuring disease activity because point reductions on the SLEDAI only occur if the organ manifestation resolves.

It is possible, mathematically, for a patient to have both improvements and exacerbations in disease activity and still meet the criteria for a responder on SELENA-SLEDAI. For example, thrombocytopenia only counts as 1 point on SELENA-SLEDAI regardless of its severity. If a patient's platelet count dropped from 99,000 to 2,000 but some other measurements improved, then that patient would be considered a responder. To compensate, the SRI built in the flare criteria on the British Isles Lupus Assessment Group instrument and the physician global assessment to gain a more accurate assessment of a patient's disease activity.

Clinical practice

What does it really mean in clinical practice to be a responder on the SRI? Simply put, it means an improvement in disease activity. It also means the patient has had a substantial reduction in severe lupus

This presentation has not been subject to peer review. The statements and opinions expressed herein are those of the author, who bears full responsibility for the content of this presentation.

Table 6. SLE Responder Index measurement used in belimumab phase III clinical trials

Testing tool	Response criteria	Instrument design
SLEDAI [24]	4-point reduction in score	Assesses 24 weighted variables
BILAG [25]	No new BILAG A	Measures flare activity across eight organs
	OR	
	Two new BILAG B organ domain scores	
Physicians Global Assessment [26]	No worsening of scores (<0.3-point increase)	Provides overall assessment of changes

BILAG, British Isles Lupus Assessment Group; SLE, systemic lupus erythematosus; SLEDAI, SLE Disease Activity Index.

flares, which can potentially improve a patient's outcomes in terms of attending work or school, and it is likely that the prednisone dose can be reduced or, at least, not increased.

Furthermore, improvements in serology matter. Patients who have high anti-DNA or low complement are twice as likely to flare during the next year, although they are not necessarily going to flare during the next week or month. Also, improvements in quality of life are important. These criteria matter to patients.

These parameters also matter to clinical researchers. Although the requirements for SRI response in clinical trials are more stringent, the goal is the same – to accurately measure clinical parameters that provide data to gauge clinically important improvements in the patient's SLE disease activity.

ABBREVIATIONS

ACR, American College of Rheumatology; CV, cardiovascular; ELISA, enzyme-linked immunosorbent assay; HCQ, hydroxychloroquine; NSAID, nonsteroidal anti-inflammatory drug; SELENA-SLEDAI, Safety of Estrogens in Lupus Erythematosus National Assessment – SLE Disease Activity Index; SLE, systemic lupus erythematosus; SLICC, Systemic Lupus International Collaborating Clinics; SRI, SLE Responder Index.

REFERENCES

1. Petri M, Orbai AM, Alarcón GS, Gordon C, Merrill JT, Fortin PR, Bruce IN, Isenberg D, Wallace DJ, Nived O, Sturfelt G, Ramsey-Goldman R, Bae SC, Hanly JG, Sánchez-Guerrero J, Clarke A, Aranow C, Manzi S, Urowitz M, Gladman D, Kalunian K, Costner M, Werth VP, Zoma A, Bernatsky S, Ruiz-Irastorza G, Khamashta MA, Jacobsen S, Buyon JP, Maddison P, *et al*.: **Derivation and validation of the Systemic Lupus International Collaborating Clinics classification criteria for systemic lupus erythematosus.** *Arthritis Rheum* 2012, **64:**2677-2686.
2. Thamer M, Hernan MA, Zhang Y, Cotter D, Petri M: **Prednisone, lupus activity, and permanent organ damage.** *J Rheumatol* 2009, **36:**560-564.
3. Magder LS, Petri M: **Incidence of and risk factors for adverse cardiovascular events among patients with systemic lupus erythematosus.** *Am J Epidemiol* 2012, **176:**708-719.
4. Danowski A, Magder L, Petri M: **Flares in lupus: outcome assessment trial (FLOAT), a comparison between oral methylprednisolone and intramuscular triamcinolone.** *J Rheumatol* 2006, **33:**57-60.
5. Hill D, Egger P, Fu Q, Fang H, Petri M: **Systemic lupus erythematosus disease activity during a 12-month period and risk of new onset organ system damage and/or death: observations in a single US academic medical center.** *Arthritis Rheum* 2011, **63(Suppl 10):**S671-S672.
6. Canadian Hydroxychloroquine Study Group: **A randomized study of the effect of withdrawing hydroxychloroquine sulfate in systemic lupus erythematosus.** *N Engl J Med* 1991, **324:**150-154.
7. Fessler BJ, Alarcon GS, McGwin G, Roseman J, Bastian HM, Friedman AW, Baethge BA, Vila L, Reveille JD; LUMINA Study Group: **Systemic lupus erythematosus in three ethnic groups: XVI. Association of hydroxychloroquine use with reduced risk of damage accrual.** *Arthritis Rheum* 2005, **52:**1473-1480.
8. Wallace DJ, Metzger AL, Stecher VJ, Turnbull BA, Kern PA: **Cholesterol-lowering effect of hydroxychloroquine in patients with rheumatic disease: reversal of deleterious effects of steroids on lipids.** *Am J Med* 1990, **89:**322-326.
9. Petri M: **Hydroxychloroquine use in the Baltimore Lupus Cohort: effects on lipids, glucose and thrombosis.** *Lupus* 1996, **5(Suppl 1):**S16-S22.
10. Pierangeli SS, Harris EN: **In vivo models of thrombosis for the antiphospholipid syndrome.** *Lupus* 1996, **5:**451-455.
11. Petri M: **Thrombosis and systemic lupus erythematosus: the Hopkins Lupus Cohort perspective.** *Scand J Rheumatol* 1996, **25:**191-193.
12. Ruiz-Irastorza G, Ramos-Casals M, Brito-Zeron P, Khamashta MA: **Clinical efficacy and side effects of antimalarials in systemic lupus erythematosus: a systematic review.** *Ann Rheum Dis* 2010, **69:**20-28.
13. Kasitanon N, Fine DM, Haas M, Magder LS, Petri M: **Hydroxychloroquine use predicts complete renal remission within 12 months among patients treated with mycophenolate mofetil therapy for membranous lupus nephritis.** *Lupus* 2006, **15:**366-370.
14. Gladman DD, Ibanez D, Urowitz MB: **Clinical impact of frequency of visits in systemic lupus erythematosus [abstract 2301].** *Arthritis Rheum* 2011, **63(10 Suppl):**S899.
15. Tench CM, McCurdie I, White PD, D'Cruz DP: **The prevalence and associations of fatigue in systemic lupus erythematosus.** *Rheumatology* (Oxford) 2000, **39:**1249-1254.
16. Wang B, Gladman DD, Urowitz MB: **Fatigue in lupus is not correlated with disease activity.** *J Rheumatol* 1998, **25:**892-895.
17. Gladman DD, Urowitz MB, Gough J, MacKinnon A: **Fibromyalgia is a major contributor to quality of life in lupus.** *J Rheumatol* 1997, **24:**2145-2148.
18. Wolfe F, Ross K, Anderson J, Russell IJ, Hebert L: **The prevalence and characteristics of fibromyalgia in the general population.** *Arthritis Rheum* 1995, **38:**19-28.
19. Wang C, Schmid CH, Rones R, Kalish R, Yinh J, Goldenberg DL, Lee Y, McAlindon T: **A randomized trial of tai chi for fibromyalgia.** *N Engl J Med* 2010, **363:**743-754.
20. Tench CM, McCarthy J, McCurdie I, White PD, D'Cruz DP: **Fatigue in systemic lupus erythematosus: a randomized controlled trial of exercise.** *Rheumatology* 2003, **42:**1050-1054.
21. Furie RA, Petri MA, Wallace DJ, Ginzler EM, Merrill JT, Stohl W, Chatham WW, Strand V, Weinstein A, Chevrier MR, Zhong ZJ, Freimuth WW: **Novel evidence-based systemic lupus erythematosus responder index.** *Arthritis Rheum* 2009, **61:**1143-1151.
22. Wells GA, Tugwell P, Kraag GR, Baker PR, Groh J, Redelmeier DA: **Minimum important difference between patients with rheumatoid arthritis: the patient's perspective.** *J Rheumatol* 1993, **20:**557-560.
23. Petri M, Hellmann D, Hochberg M: **Validity and reliability of lupus activity measures in the routine clinical setting.** *J Rheumatol* 1992, **19:**53-59.
24. Bombardier C, Gladman DD, Urowitz MB, Caron D, Chang CH:

This presentation has not been subject to peer review. The statements and opinions expressed herein are those of the author, who bears full responsibility for the content of this presentation.

Derivation of the SLEDAI: a disease activity index for lupus patients. *Arthritis Rheum* 1992, **35:**630-640.
25. Hay EM, Bacon PA, Gordon C, Isenberg DA, Maddison P, Snaith ML, Symmons DP, Viner N, Zoma A: **The BILAG index: a reliable and valid instrument for measuring clinical disease activity in systemic lupus erythematosus.** *Q J Med* 1993, **86:**447-458.
26. Navarra SV, Guzmán RM, Gallacher AE, Hall S, Levy RA, Jimenez RE, Li EK, Thomas M, Kim HY, León MG, Tanasescu C, Nasonov E, Lan JL, Pineda L, Zhong ZJ, Freimuth W, Petri MA; BLISS-52 Study Group: **Efficacy and safety of belimumab in patients with active systemic lupus erythematosus: a randomised, placebo-controlled, phase 3 trial.** *Lancet* 2011, **377:**721-731.

This presentation has not been subject to peer review. The statements and opinions expressed herein are those of the author, who bears full responsibility for the content of this presentation.
